# Small Peptide Inhibitor of JNK3 Protects Dopaminergic Neurons from MPTP Induced Injury via Inhibiting the ASK1-JNK3 Signaling Pathway

**DOI:** 10.1371/journal.pone.0119204

**Published:** 2015-04-09

**Authors:** Jing Pan, Hui Li, Bei Zhang, Ran Xiong, Yu Zhang, Wen-Yan Kang, Wei Chen, Zong-Bo Zhao, Sheng-Di Chen

**Affiliations:** 1 Department of Neurology and Institute of Neurology, Ruijin Hospital Affiliated to Shanghai Jiao Tong University School of Medicine, Shanghai, China; 2 Key Laboratory of Stem Cell Biology & Laboratory of Neurodegenerative Diseases, Institute of Health Science, Shanghai Institutes of Biological Sciences (SIBS), Chinese Academy of Science (CAS) and Shanghai Jiao Tong University School of Medicine, Shanghai, China; Department of Pathology, Anatomy & Cell Biology, Thomas Jefferson University, UNITED STATES

## Abstract

**Introduction and Aims:**

The ASK1-JNK3 signaling pathway plays a pivotal role in the pathogenesis of Parkinson's disease (PD). The specific binding of β-arrestin2 to JNK3 is essential for activation of the ASK1-JNK3 cascade, representing a potential therapeutic target for preventing dopaminergic neuronal death in PD. The aim of this study was to identify a novel strategy for the prevention of dopaminergic neuronal death in PD.

**Methods:**

Based on the specific binding of β-arrestin2 to JNK3, a 21-amino-acid fusion peptide, termed JNK3-N-Tat, was synthesized. We evaluated the ability of this peptide to inhibit the binding of β-arrestin2 to its target domain in JNK3 in vitro and in vivo.

**Results:**

The JNK3-N-Tat peptide inhibited activation of the ASK1-JNK3 cascade by disrupting the interaction between β-arrestin2 and JNK3. JNK3-N-Tat exerted beneficial effects through pathways downstream of JNK3 and improved mitochondrial function, resulting in attenuated MPP+/MPTP-induced damage. JNK3-N-Tat protected mesencephalic dopaminergic neurons against MPTP-induced toxicity.

**Conclusions:**

JNK3-N-Tat, a JNK3-inhibitory peptide, protects dopaminergic neurons against MPP+/MPTP-induced injury by inhibiting the ASK1-JNK3 signaling pathway.

## Introduction

Parkinson’s disease (PD) is a neurodegenerative disorder that affects approximately 1% of people over the age of 65 and 2.6% of people over the age of 85[1]. The characteristic pathological effects of PD are the selective and progressive loss of dopaminergic neurons in the substantia nigra pars compacta (SNc) of the midbrain and the formation of Lewy bodies in surviving dopaminergic cells[2]. A growing body of evidence indicates that the strong activation of c-Jun N-terminal protein kinases (JNKs), and JNK3 in particular, are involved in the molecular mechanisms of selective dopaminergic neuronal death in PD. Our previous study revealed two peaks of JNK3 activation in dopaminergic neurons after MPTP-based injury [3]. Furthermore, Choi and colleagues discovered that JNK3 is the common critical mediator of dopaminergic neuronal death induced by both paraquat and rotenone [4].

Together with JNK1 and JNK2, JNK3 belongs to the JNK family of kinases and acts as a key regulator of stress-induced apoptosis[5]. The three JNKs isoforms differ in their tissue expression profiles and functions. JNK1 and JNK2 are ubiquitously expressed, whereas JNK3 is expressed most strongly in the brain, as well as at lower levels in the heart and the testes [6,7]. Apoptosis signal-regulating kinase 1 (ASK1), a positive regulator of JNK3, can be turned on in response to diverse stressors and apoptotic stimuli, leading to activation the ASK1-JNK3 signaling pathway[8]. Activated JNK3 phosphorylates a variety of target proteins, including nuclear factors and mitochondrial proteins, leading to apoptosis and mitochondrial dysfunction[9]. β-arrestin2 is a scaffold protein and a key mediator of ASK1-JNK3 signaling pathway activation. The results of a study by Song and colleagues showed that β-arrestin2 is the only isoform of the four mammalian arrestins that facilitates JNK3 phosphorylation [10]. β-arrestin2 assembles three components of the ASK1-MKK4-JNK3 signaling complex to promote signal compartmentalization. Additional detailed studies identified the key sites necessary for the binding of JNK to β-arrestin2, which are located in the N-terminus of JNK3 [10,11,12]. The binding of β-arrestin2 to ASK1-JNK3 may regulate apoptosis in adult neurons, making this signaling pathway a potential therapeutic target for drug development [10,13]. It is possible that even subtle changes to the binding of β-arrestin2 to the ASK1-MKK4-JNK3 complex could hinder the proper conformational and functional interactions between these proteins, thereby preventing JNK3 activation. Therefore, the ASK1-JNK3 signaling pathway is a potential therapeutic target for the prevention of dopaminergic neuronal death in PD.

Based on the above information, we hypothesized that blocking access of the scaffold protein β-arrestin2 to its JNK3 target domain (amino acids C9 and I18 in the N-terminus) could allow us to regulate the activity of the ASK1-JNK3 cascade and, in turn, the apoptosis of dopaminergic neurons. Therefore, we generated a 21-amino-acid fusion peptide, termed JNK3-N-Tat, and characterized its function as a JNK3 inhibitor *in vitro* and *in vivo*.

## Materials and Methods

### Antibodies and reagents

The following antibodies were used: rabbit polyclonal anti-ASK1 (Santa Cruz, sc-7931); rabbit polyclonal anti-phospho-ASK1 (Thr845) (Millipore, 3765); rabbit monoclonal anti-JNK3/SAPK1b, clone C05T (Millipore, 04–893); anti-JNK3 (4G6) (Santa Cruz, sc-81469); rabbit polyclonal anti-tyrosine hydroxylase (Millipore, AB152), mouse monoclonal anti-p-JNK (G-7) IgG (Santa Cruz, sc-6254); goat polyclonal anti-β-arrestin2 (n-16) (Santa Cruz, sc-6386); rabbit polyclonal anti-c-Jun (H-79) IgG (Santa Cruz, sc-1694); mouse monoclonal anti-p-c-Jun (Ser63/73) (Santa Cruz, sc-6252); and Alexa Fluor 488/546 donkey anti-goat IgG (Invitrogen, A11056/A11057). All chemicals were purchased from Sigma-Aldrich, unless noted otherwise.

### Peptides treatment

The peptides used in this study were synthesized by Sango Biotech. The peptide sequences were as follows: JNK3-N-Tat, Tyr-Gly-Arg-Lys-Lys-Arg-Arg-Gln-Arg-Arg-Arg-Cys-Ser-Glu-Pro-Thr-Leu-Asp-Val-Lys-Ile; JNK3-4A-Tat, Tyr-Gly-Arg-Lys-Lys-Arg-Arg-Gln-Arg-Arg-Arg-Cys-Ser-Glu-Ala-Thr-Ala-Ala-Val-Lys-Ala. The peptides were dissolved in saline to create a 20 mM stock solution.

### Cell culture and treatments

SH-SY5Y cells were maintained in DMEM containing 10% FBS and 100 U/ml penicillin/streptomycin. To induce a dopaminergic phenotype, SH-SY5Y cells were treated with 10 μM retinoic acid (RA) in supplemented medium for 6 days. All cell culture materials were purchased from Invitrogen. Primary cultured mouse cortical neurons were prepared as described previously[14]. The cells were incubated in MPP^+^ as indicated. In addition, the cells were pretreated with either JNK3-N-Tat or JNK3-4A-Tat (20 μM) in saline for 1 hr prior to MPP^+^ treatment. The cells were harvested at the indicated time points for further analysis.

### Animals and treatments

Animal experiments were performed according to the NIH Guide for the Care and Use of Laboratory Animals and approved by the Shanghai Jiao Tong University School of Medicine Animal Care and Use Committee (2009087). Eight-week-old male C57BL/6 mice (20–25 g) were provided by the Shanghai Institutes of Biological Sciences animal center. The mice were housed 5 to a cage and provided with food and clean water ad libitum. All animals were maintained in a temperature-controlled environment (22–25°C, 40–60% relative humidity) with a 12 hr light-dark cycle. The groups of mice (n = 6) were injected intraperitoneally (i.p.) five times (one injection per day for 5 consecutive days) with 30 mg/kg/day MPTP•HCl (Sigma), which induces a reliable mouse model of PD; controls were injected with a corresponding volume of saline. The peptide-treated groups were injected i.p. with either JNK3-N-Tat or JNK3-4A-Tat (1 μg/g body weight per day) in saline either one day before or 1 hr before each administration of MPTP.

### Western blotting

Cells or tissues from mice were lysed in RIPA buffer (50 mM Tris•HCl pH 8.0, 150 mM NaCl, 1% NP-40, 0.5% sodium deoxycholate, and 0.1% SDS) containing protease inhibitor cocktail (Roche) and 1 mM phenylmethylsulfonyl fluoride for 30 min on ice. Total extracts were centrifuged at 14,000×g for 30 min. The NE-PER Nuclear and Cytoplasmic Extraction Reagent Kit was used to isolate nuclei and prepare protein extracts (Thermo Scientific), and protein concentration was determined using the BCA protein assay kit (Thermo Scientific). The samples were separated using SDS-PAGE and electrotransferred to a polyvinylidene difluoride membrane using a semidry blotting system. The proteins were detected using immunoblotting (IB) analysis with the indicated antibodies and Immobilon Western Chemiluminescent HRP Substrate (Millipore). Band intensities were quantified by densitometric analyses using the ImageJ (NIH) software program. All experiments were performed in triplicate.

### Co-immunoprecipitation (Co-IP)

Cells or tissues from mouse brains were lysed in non-denaturing lysis buffer containing 20 mM Tris HCl pH 8.0, 137 mM NaCl, 10% glycerol, 1% Nonidet P-40, 2 mM EDTA and protease inhibitors. The lysates were pre-cleared with protein A sepharose (GE bioscience) for 30 min. The supernatants were incubated in primary antibody overnight at 4°C. Then, protein A sepharose (GE Bioscience) was added, and the mixture was incubated for another 4 hr at 4°C. The beads containing the bound proteins were washed 4 times with non-denaturing lysis buffer and boiled in 2× SDS sample buffer. The samples were then examined using Western blotting analysis.

### Immunofluorescence microscopy

Cells were seeded on poly-L-lysine-coated cover slips, fixed with 4% PFA for 20 min, permeabilized with 0.2% Triton X-100 for 15 min, and then blocked with 3% BSA in 0.1% PBST for 1 hr. Next, the cells were incubated with primary antibodies against T-JNK3 and β-arrestin2 overnight at 4°C, followed by washing with PBST. The cells were then incubated with the Alexa Flour 488 and 546 IgG (Invitrogen) secondary antibodies for 30 min. Anti-fade mounting medium with DAPI (Vector Laboratory) was added, and the stained cells were analyzed by confocal microscopy (Leica SP5).

### Transmission electron microscopy

The neurons were washed in cold PBS and pre-fixed with pre-chilled 2% glutaraldehyde for 2 hr. The cells were then collected by centrifugation and post-fixed with 1% osmium tetroxide. Next, the samples were rinsed with phosphate buffer and dehydrated in an increasing ethanol gradient. The samples were embedded in Epon 812. Thin sections were prepared using an Ultracut R microtome (LEICA), stained with uranyl acetate and lead citrate, and then visualized using a CM120 transmission electron microscope (Philips).

### Apoptosis and cell viability assays

Cells were seeded into 6-well plates and treated as described above. Subsequently, the cells were stained for Annexin V using the Annexin V-FITC apoptosis detection kit (BD Bioscience), according to the manufacturer’s instructions, and analyzed using a flow cytometer (Becton Dickinson; LSR II). The effects of the two peptides on SH-SY5Y cell viability were evaluated using the cell proliferation reagent MTT (Sigma). All experiments were performed in triplicate.

### Immunohistochemistry and TH-positive cell quantification

At 7 days post-MPTP injection, animals were deeply anesthetized with pentobarbital (100 mg/kg, i.p.), and then perfused transcardially with 0.9% saline followed by 4% PFA in 100 mM phosphate buffer (PB, pH 7.4). The brains were removed by dissection, post-fixed for 24 hr with 4% PFA in PB, and placed in 30% sucrose solution in PB for 24–72 hr at 4°C. The brains were cryosectioned coronally into 12 μm sections using a CM1650 cryostat (Leica). Serial sections were collected throughout the SNc and the striatum. Adhered sections were fixed with ice-cold acetone for 15 min at -20°C and washed 3 times with PBS. The tissues were blocked with 5% goat serum in PBS for 1 hr at room temperature, followed by incubation with an anti-TH primary antibody overnight at 4°C. The sections were then incubated with the biotinylated secondary antibody overnight, followed by incubation with avidin-conjugated horseradish peroxidase for 1 hr at 37°C. Finally, the sections were incubated with the peroxidase substrate diaminobenzidine. The total number of TH-positive neurons in the SNc was counted according to the method of Furuya et al.[15] using the ‘Image Tool’ from The University of Texas Health Science Center at San Antonio (UTHSCSA); measurements were made by researchers who were blinded to the treatment conditions[16]. The optical fractionator technique with the aid of the StereoInvestigator software was using to evalutae the TH-positive fibers in striatum.

### Statistical analysis

Statistical analysis of the data was performed using the SPSS 16.0 software program. All data are expressed as mean±standard deviation of the mean values. One-way ANOVA was used to compare the differences between the experimental groups and control group. Differences were considered to be significant if the corresponding *P* values were less than 0.05. All analyses were performed while blinded to the experimental conditions.

## Results

### 1. Activation of the ASK1-JNK3 signaling pathway represents a cellar model of PD.

**Fig 1:**
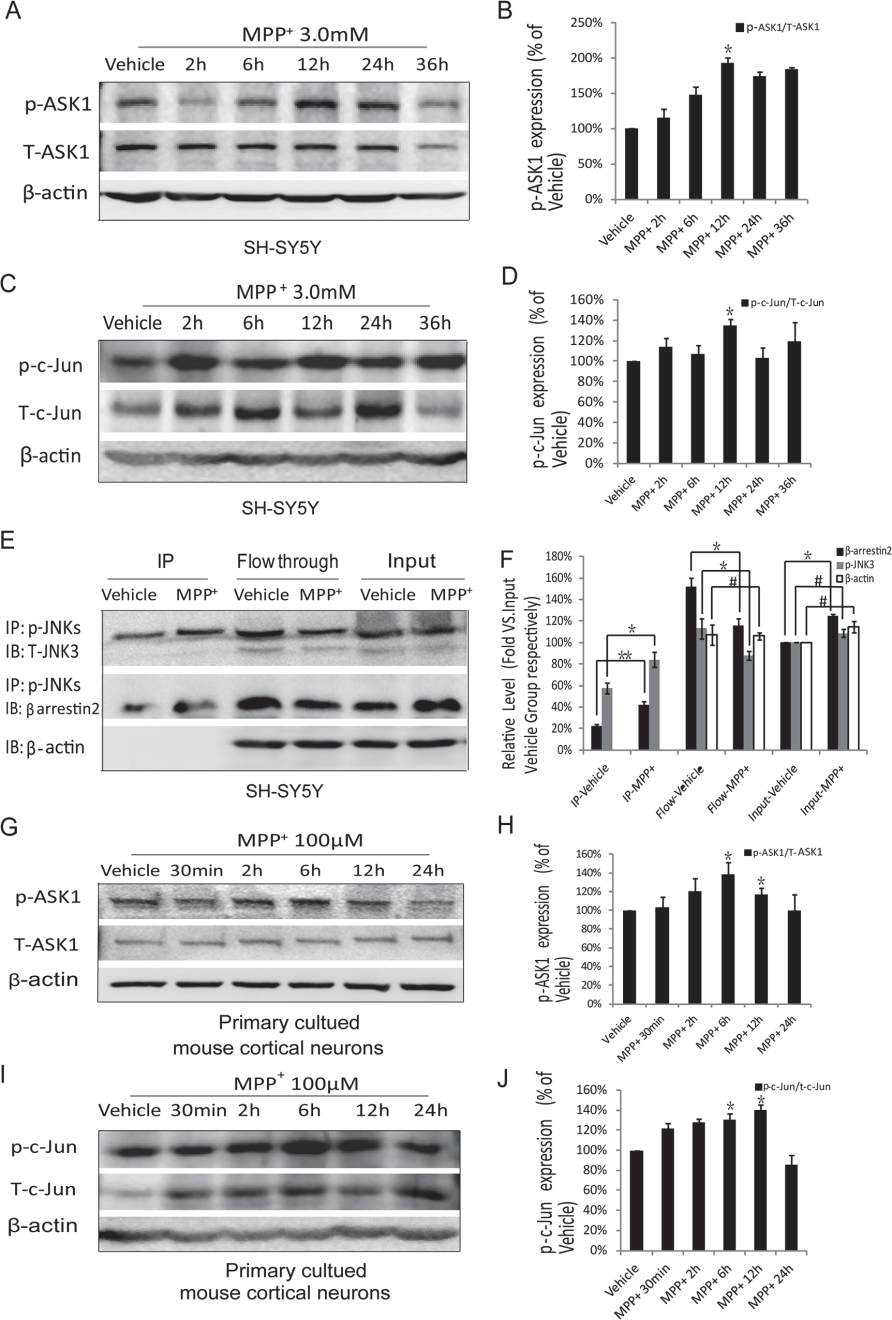
Activation of the ASK1-JNK3 signaling pathway and binding of β-arrestin2 to JNK3 were increased in MPP^+^-treated cells. SH-SY5Y cells were treated with MPP^+^ (3 mM) or vehicle for the indicated times. (A, C) Western blotting analysis of p-ASK1, T-ASK1, p-c-Jun and T-c-Jun in extracts from SH-SY5Y cells after MPP^+^ treatment. (E) p-JNK3 was examined using IP and IB analyses in lysates from SH-SY5Y cells treated with MPP^+^ for 12 hr. The results revealed that p-JNK3 expression was increased and that the binding of β-arrestin2 to JNK3 was significantly increased. (G, I) Neurons were treated with MPP^+^ (100 μM) or vehicle for the indicated times. Western blotting analysis of p-ASK1, T-ASK1, p-c-Jun and T-c-Jun in extracts from cells after MPP^+^ treatment. (B, D, F, H, J) Bar graphs show the results of our statistical analysis (mean±SD) from three independent experiments. Flow: Flow-through; Input: Supernatant of non-denaturing lysis buffer. * *P*<0.05; ** *P*<0.01; # *P*>0.05.

A critical role for ASK1 in 6-hydroxydopamine (6-OHDA)-induced apoptosis in the SH-SY5Y human neuroblastoma cell line has been described[17]. Our previous study demonstrated the activation of ASK1 in a 6-OHDA-induced PD animal model[3]. Furthermore, recent studies have revealed the involvement of ASK1 in L-DOPA-induced neuronal apoptosis in a cellar model of PD [18]. However, the effects of MPP^+^/MPTP on ASK1 and the ASK1-JNK3 pathway have not been described. To create the proper conditions for our study, SH-SY5Y cells were incubated in MPP^+^ as a cellar model for PD. We found that ASK1 activity was increased in SH-SY5Y cells following MPP^+^ (3 mM) treatment and that the peak of ASK1 activation was after 12 hr (Fig 1A and Fig 1B). Using phospho-specific antibodies against c-Jun, we found that exposure to MPP^+^ for 12 hr resulted in an increase in p-c-Jun immunoreactivity (Fig 1C and Fig 1D). These data indicated that phosphorylation of the upstream kinase (p-ASK1) and downstream substrates (p-c-Jun) of JNK3 were dramatically increased 12 hr after treatment with MPP^+^. Next, we examined the interaction between β-arrestin2 and JNK3 using co-IP analysis in SH-SY5Y cells treated with MPP^+^ for 12 hr. The results revealed that p-JNK3 expression was increased by approximately 50% under the experimental conditions and that β-arrestin2 binding to JNK3 was significantly increased (Fig 1E and Fig 1F). These results demonstrate aberrant activation of the ASK1-JNK3 signaling pathway and increased binding of β-arrestin2 to JNK3 in the MPP^+^-induced SH-SY5Y cellar model of PD.

Activation of the ASK1-JNK3 pathway was also detected in primary cultured mouse cortical neurons that were incubated with MPP^+^ (100 μM). We also observed an increase in the level of p-ASK1 in lysates from neurons treated with MPP^+^ (Fig 1G and Fig 1H), and c-Jun phosphorylation was up regulated by MPP^+^-induced injury (Fig 1I and Fig 1J). Moreover, the neuronal data clearly show effects on the upstream kinase and downstream substrates of JNK3 6 hr after MPP^+^ treatment.

These results show that ASK1-JNK3 is a critical pathway involved in dopaminergic neuronal death under MPP^+^-induced injury conditions. Therefore, we thought it might be possible to inhibit the ASK1-JNK3 pathway by targeting the binding of β-arrestin2 to JNK3.

### 2. Visualization of the intra-neuronal accumulation of JNK3-N-Tat-Dansyl.

**Fig 2:**
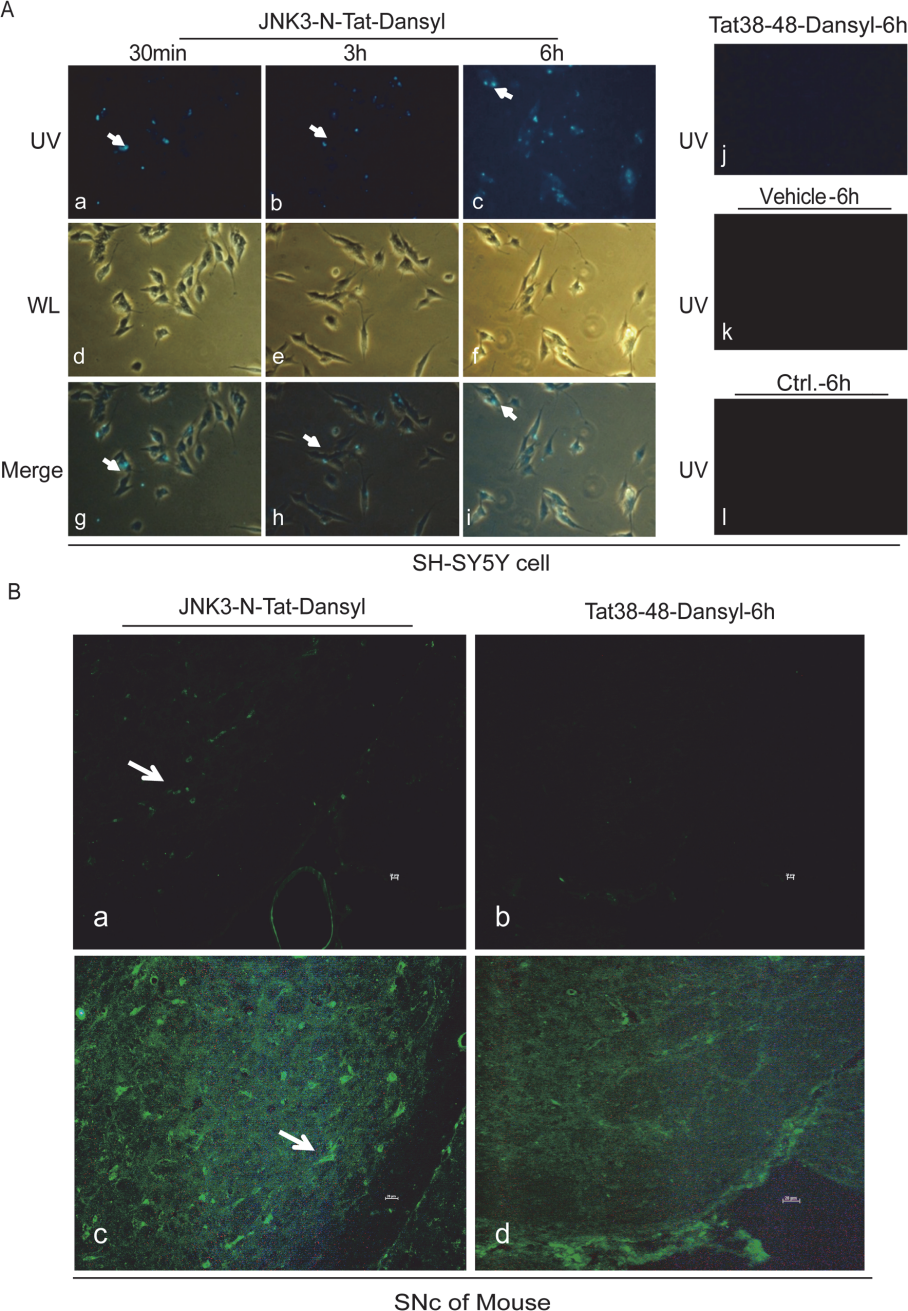
JNK3-N-Tat-Dansyl, but not Tat38-48-Dansyl, accumulates within neurons. (A) SH-SY5Y cells were treated with JNK3-N-Tat-Dansyl (a-i), Tat38-48-Dansyl (j) or vehicle (k) for the indicated periods. JNK3-N-Tat-Dansyl (20 μM), but not Tat38-48-Dansyl (20 μM), exhibited strong fluorescence under UV light 30 min after application to SH-SY5Y cell cultures (40× magnification). The arrows indicate co-localization of JNK3-N-Tat-Dansyl with the SH-SY5Y cell body. WL: white light. (B) Fluorescence was detected under UV light in coronal sections from the SNc (Fig. B, a and c) of mice injected i.p. with JNK3-N-Tat-Dansyl (1 μg/g.kw). Arrows indicate the localization of JNK3-N-Tat-Dansyl in the SNc. n = 3. Scale bars in B (a–d): 1 = 20 μm.

Based on the above results, we attempted to develop a specific neuroprotective therapy to protect dopaminergic neurons against MPP^+^-induced injury by disturbing the binding of β-arrestin2 to JNK3. We synthesized a 21-amino acid fusion peptide, termed JNK3-N-Tat. This peptide is composed of two parts: (1) a 10-mer peptide identical to the N-terminal of JNK3, and (2) the Tat peptide from the cell-membrane transduction domain of HIV-1, which allows JNK3-N-Tat to penetrate cells. A mutant version of the peptide, termed JNK3-4A-Tat, was used as a negative control, and it contained four-point mutations in the DNA sequence of JNK3. To measure the cell permeability of JNK3-N-Tat, the peptide was conjugated to the fluorophore dansyl chloride, referred to as JNK3-N-Tat-Dansyl. Tat38-48-Dansyl was used as a negative control. SH-SY5Y cells treated with JNK3-N-Tat-Dansyl exhibited green fluorescence in the cell body (Fig 2A.a-I. However, no fluorescence was detected in cells treated with Tat38-48-Dansyl or vehicle alone under ultraviolet laser excitation (Fig 2A.j-l). Next, we examined the cell permeability of these peptides in mice injected i.p. with JNK3-N-Tat-Dansyl or Tat38-48-Dansyl. Fluorescence was detected in coronal sections of the SNc of mice injected with JNK3-N-Tat-Dansyl, although only background signal was detected in the same region of mice treated with Tat38-48-Dansyl (Fig 2B. Therefore, these results indicated that the membrane transduction domain of HIV-1 effectively facilitated the entry of JNK3-N-Tat into cells of the SNc.

### 3. JNK3-N-Tat attenuates MPP^+/^MPTP-induced activation of JNK3 by inhibiting the binding of β-arrestin2 to JNK3 following MPP^+^/MPTP-induced injury.

**Fig 3:**
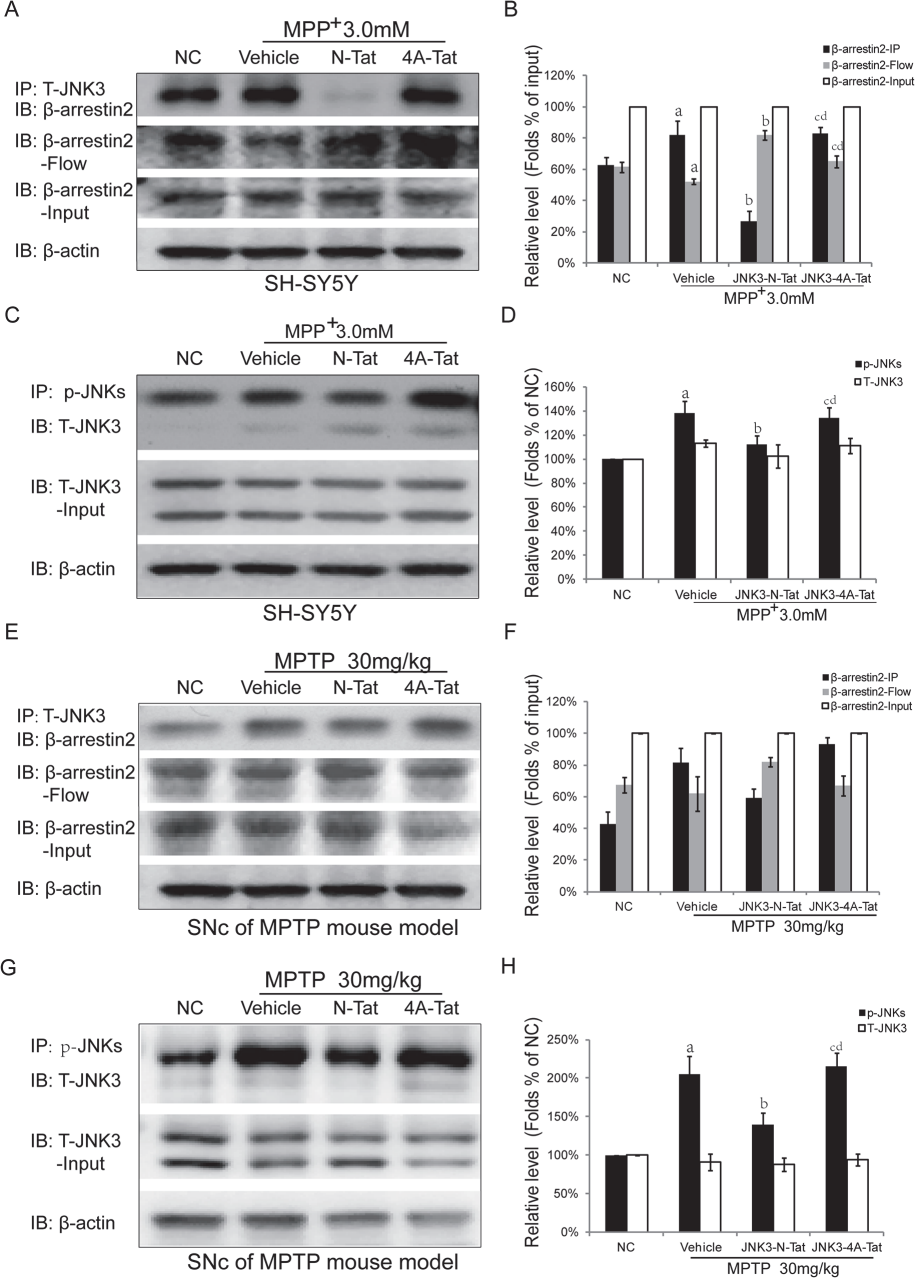
JNK3-N-Tat attenuated the MPP^+/^MPTP-induced activation of JNK3 by inhibiting the binding of β-arrestin2 to JNK3 following MPP^+^-induced injury. SH-SY5Y cells were pretreated with vehicle, JNK3-N-Tat, or JNK3-4A-Tat 1 h before MPP^+^ (3 mM) treatment and then cultured for 12 hr before analysis. Mice from the normal control group were subjected to sham treatment, and mice in the other three groups were administered saline vehicle, JNK3-N-Tat, or JNK3-4A-Tat one day before MPTP injection and then 1 h before each MPTP injection for 5 subsequent days. (A, C) In the celarl model of PD, co-immunoprecipitation analysis revealed that JNK3-N-Tat attenuated the MPP^+^-induced activation of JNK3 by inhibiting the binding of β-arrestin2 to JNK3. (E, G) In the MPTP mouse model, the results revealed that JNK3-N-Tat treatment decreased the MPTP-induced activation of JNK3 and inhibited the binding of β-arrestin2 to JNK3 (n = 6 animals per group). (B, D, F, H) Bar graphs display the results of our statistical analysis (mean±SD). NC: Normal control; N-Tat: JNK3-N-Tat; 4A-Tat: JNK3-4A-Tat. Flow: Flow-through; Input: Supernatant of non-denaturing lysis buffer. a: *P*<0.05 versus Normal control; #: *P*>0.05 versus Normal control; b: *P*<0.05 versus vehicle; c: *P*<0.05 versus JNK3-N-Tat; d: *P*>0.05 versus vehicle.

To evaluate the effects of the JNK3 peptides on the JNK3 cascade, JNK3-N-Tat or JNK3-4A-Tat (20 μM) was administered 1 hr before MPP^+^ insult. Co-IP analysis revealed that treatment with JNK3-N-Tat, but not JNK3-4A-Tat, inhibited the binding between JNK3 and β-arrestin2 (Fig 3A and Fig 3B) and attenuated the MPP^+^-induced activation of JNK3 (Fig 3C and Fig 3D). Next, we administered JNK3-N-Tat or JNK3-4A-Tat to mice before MPTP treatment. The results indicated that JNK3-N-Tat decreased the binding between JNK3 and β-arrestin2 (Fig 3E and Fig 3F) in the MPTP mouse model. Consistent with the above results, JNK3-N-Tat attenuated the activation of JNK3 in the MPTP mouse model. In contrast, treatment with JNK3-4A-Tat did not attenuate the detrimental effects induced by MPP^+^ or MPTP (Fig 3A–3H). The effect of JNK3-N-Tat on the JNK3 cascade was also analyzed in neurons treated with MPP^+^ (100 μM). As shown in Fig 3A, MPP^+^ clearly induced the co-localization of JNK3 and β-arrestin2. However, the MPP^+^-induced co-localization of JNK3 and β-arrestin2 was significantly decreased in the group that was pretreated with JNK3-N-Tat (Fig 3A.iii). These data suggest that JNK3-N-Tat attenuated MPP^+/^MPTP-induced activation of JNK3 by inhibiting the binding of β-arrestin2 to JNK3 following MPP^+/^MPTP-induced injury. β-arrestin2 protein expression levels are not changed after MPP+ injury (Data not shown).

### 4. The effect of JNK3-N-Tat on the downstream effectors of JNK3 in a PD model.

**Fig 4:**
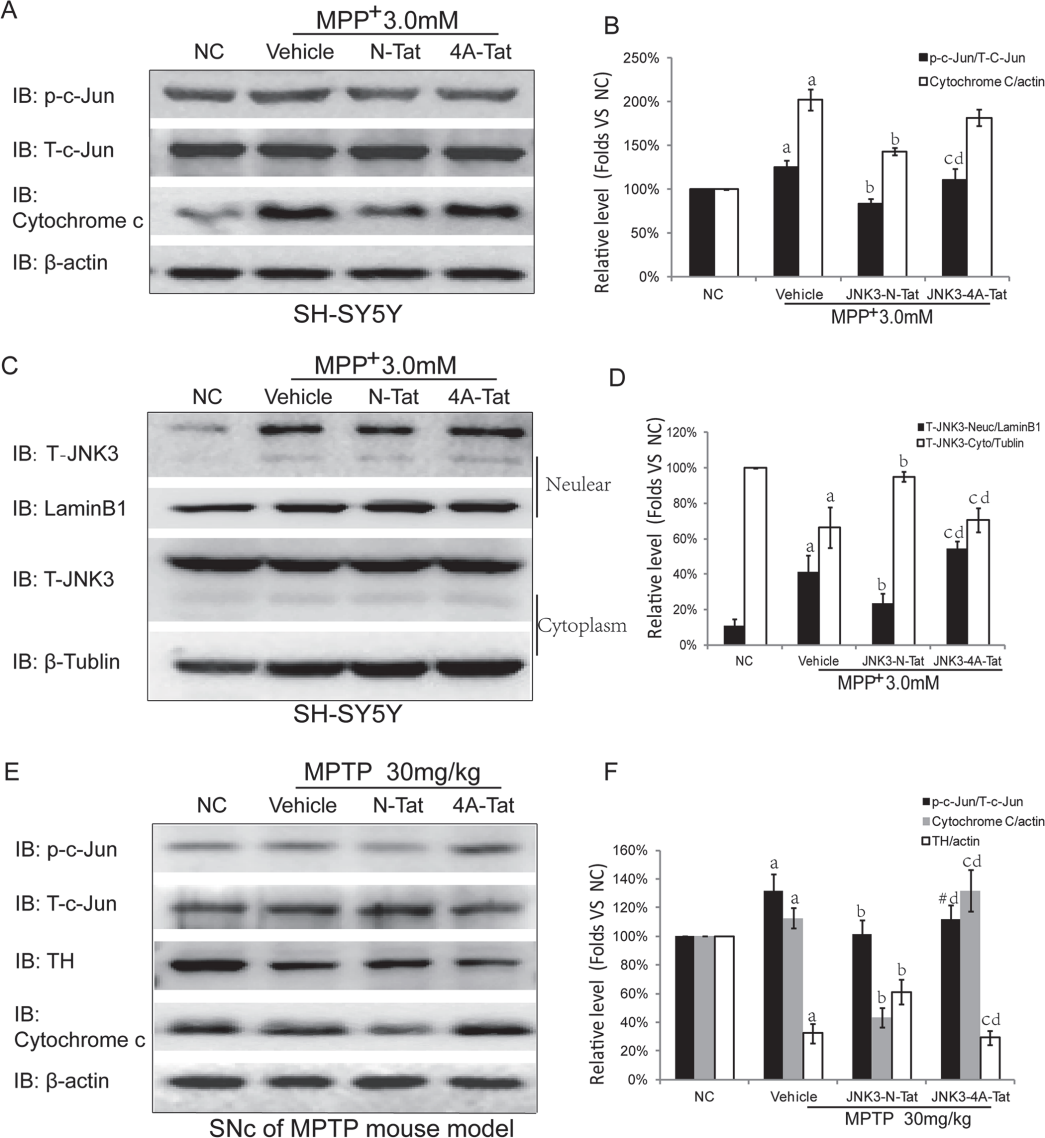
Effects of JNK3-N-Tat on the downstream effectors of JNK3 in PD models. (A) Western blotting analysis of p-c-Jun, T-c-Jun and cytochrome c in SH-SY5Y cell extracts after treatment. The expression levels of cytochrome c and p-c-Jun in the JNK3-N-Tat-treated groups were clearly decreased compared with the MPP^+^- and JNK3-4A-Tat-treated groups. (C) Cytosolic and nuclear fractions were separated, and nuclear and cytosolic T-JNK3 levels were assessed by Western blotting. The results revealed decreased nuclear translocation of T-JNK3 in the JNK3-N-Tat group compared with the vehicle group. Treatment with JNK3-N-Tat did not result in JNK3 returning to the cytoplasm. (E) Western blotting analysis of p-c-Jun, T-c-Jun, cytochrome c and TH in extracts from mouse SNc. The expression of TH in JNK3-N-Tat-treated mice was greater than in MPTP- or JNK3-4A-Tat-treated mice. JNK3-N-Tat treatment also attenuated the MPP^+^-induced activation of c-Jun and cytochrome c in the MPTP mouse model (n = 6 animals per group). (B, D, F) Bar graphs display the results of our statistical analysis (mean±SD). NC: Normal control; N-Tat: JNK3-N-Tat; 4A-Tat: Tat-4A-Tat. a: *P*<0.05 versus Normal control; b: *P*<0.05 versus vehicle; c: *P*<0.05 versus JNK3-N-Tat; d: *P*>0.05 versus vehicle; #: *P*>0.05 versus JNK3-N-Tat.

Activated JNK3 participates in many pathological processes by regulating a variety of substrates and mitochondrial proteins[19]. To further confirm the effect of JNK3-N-Tat on the JNK3 pathway, cytochrome c and proteins downstream of JNK3 were measured. As shown in Fig 4A and 4B and 4E and 4F, the expression levels of cytochrome c and p-c-Jun in the JNK3-N-Tat-treated groups were clearly decreased compared with the MPP^+^ or JNK3-4A-Tat-treated groups. In the MPTP mouse model of PD, we detected the expression of TH in the SNc. These results revealed that the expression of TH in JNK3-N-Tat-treated mice was greater than that in either MPTP- or JNK3-4A-Tat-treated mice. As reported previously, JNK3 is widely expressed in both the nucleus and cytoplasm, and activated JNK3 can translocate to the nucleus. In our study, treatment with JNK3-N-Tat reduced the expression level of nuclear JNK3 in MPP^+^-treated SH-SY5Y cells (Fig 4C and Fig 4D). Therefore, treatment with JNK3-N-Tat not only showed beneficial effects on the downstream effectors of JNK3 but also induced the retention of JNK3 in the cytoplasm in a cellar model of PD.

### 5. JNK3-N-Tat diminishes MPP^+^-induced primary cortical neuronal apoptosis and mitochondrial damage.

**Fig 5:**
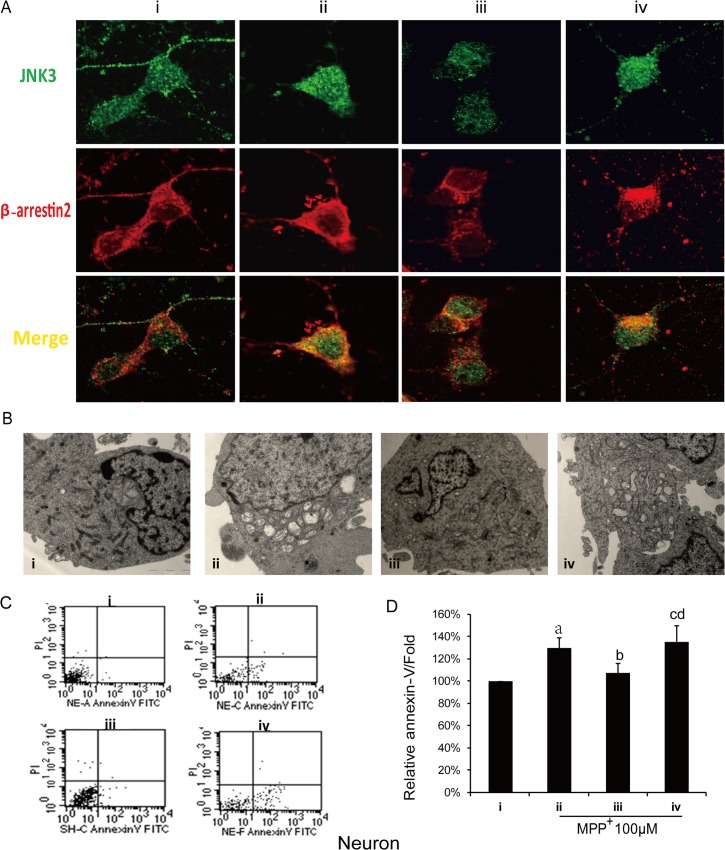
Effects of JNK3-N-Tat on mitochondrial damage and apoptosis in MPP^+^-treated primary cultured cortical neurons. Representative images of the sham-treated normal control group (i) and the groups administered saline vehicle (ii), JNK3-N-Tat (iii), or JNK3-4A-Tat (iv) before MPP^+^ treatment to cortical neurons. Pretreatment with 20 μm JNK3-N-Tat or JNK3-4A-Tat was performed 1 hr before MPP^+^ treatment (100 μM). The cells were then cultured for 6 hr, followed by fixation or staining with donkey anti-T-JNK3 and donkey anti-β-arrestin2 antibodies. (A) Co-localization of T-JNK3 and β-arrestin2 in MPP^+^-treated cortical neurons. Representative confocal images show that JNK3-N-Tat inhibited the co-localization of JNK3 and β-arrestin2 induced by MPP^+^ in primary cultured cortical neurons. (B) Electron microscopy revealed that JNK3-N-Tat treatment protected neuronal mitochondria against MPP^+^-induced injury. (C, D) Flow cytometry revealed that JNK3-N-Tat treatment attenuated the MPP^+^-induced neuronal apoptosis. Bar graphs show the results of our statistical analysis (mean±SD) from three independent experiments. a: *P*<0.05 versus Normal control; b: *P*<0.05 versus vehicle; c: *P*<0.05 versus JNK3-N-Tat; d: *P*>0.05 versus vehicle.

To further explore the effects of JNK3-N-Tat, we evaluated morphology of mitochondria and level of apoptosis in neurons. Electron microscopy was used to examine morphological changes in the groups of cells treated with or without JNK3-N-Tat. The images revealed that MPP^+^ induced the swelling and loss of mitochondria and that JNK3-N-Tat could attenuate this MPP^+^-induced mitochondrial injury in neurons (Fig 5B). Next, we used flow cytometry to evaluate apoptosis levels, and the results indicated that JNK3-N-Tat attenuated MPP^+^-induced apoptosis (Fig 5C and Fig 5D).

### 6. Effect of JNK3-N-Tat on MPTP-induced dopaminergic neuronal death in cell and animal models of PD.

**Fig 6:**
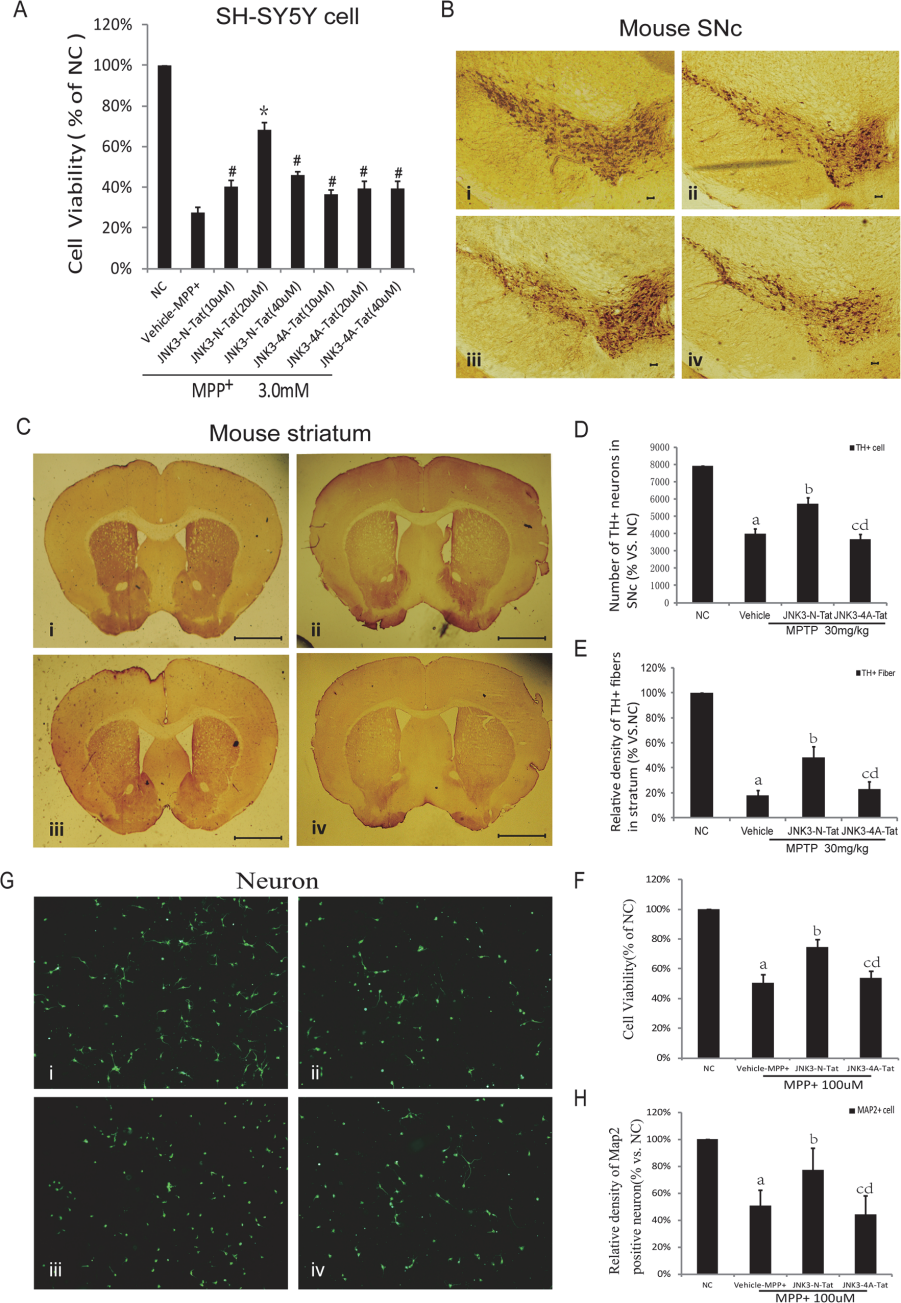
Effect of JNK3-N-Tat on MPP^+^/MPTP-induced neuronal death in PD models. (A) Pretreatment with 20 μM JNK3-N-Tat resulted in a significant attenuation of MPP^+^-induced SH-SY5Y cell death. *P<0.05 versus vehicle-MPP^+^; #*P*>0.05 versus vehicle-MPP^+^. (B, C) Animals were sacrificed 7 days post-MPTP administration to characterize the loss of dopaminergic neurons. Representative images from immunostained sections of the SNc and the striatum from the sham-treated normal control group (i), the group of mice administered saline prior to MPP^+^ treatment (ii), and the groups of mice administered JNK3-N-Tat (iii) or JNK3-4A-Tat (iv) preceding MPTP injection. The results revealed that treatment with JNK3-N-Tat caused a significant decrease in neurodegeneration in the SNc and also attenuated MPTP-induced striatal DA terminal loss, whereas treatment with JNK3-4A-Tat had no effect. Scale bars in B(i–iv): 1 = 10μm; Scale bars in C(i–iv): 1 = 100 μm. (D, E) Quantitative analysis of the protective effects of JNK3-N-Tat on MPTP-induced mesencephalic dopaminergic neuronal damage. The numbers of surviving TH-positive cells in the SNc and TH-positive fibers in the striatum were counted. The data are expressed as the mean±SD values (n = 6 animals per group). (F) In primary cultured cortical neurons, our results revealed that pretreatment with 20 μM JNK3-N-Tat 1 hr before MPP^+^(100 μM) treatment resulted in significant attenuation of cell death. (G, H) Immunofluorescence stainings using an anti-MAP2 antibody revealed that JNK3-N-Tat treatment decreased the loss of MAP2-positive neurons (20× magnification). a: *P*<0.05 versus Normal control; b: *P*<0.05 versus vehicle; c: *P*<0.05 versus JNK3-N-Tat; d: *P*>0.05 versus vehicle.

First, we evaluated the effects of the JNK3 peptides on neuronal death induced by MPP^+^ using MTT assays. As shown in Fig 6A, MPP^+^ induced roughly 70% cell death. However, pretreatment with 20 μM JNK3-N-Tat resulted in a significant reduction in cell death (30%). In contrast, treatment with the control peptide JNK3-4A-Tat did not protect cells against MPP^+^-induced injury. This result indicated a protective role for JNK-N-Tat against MPP^+^. We obtained similar results with primary cultured cortical neurons as we did with SH-SY5Y cells, as shown in Fig 6F. The results of the MTT assays revealed that pretreatment with 20 μM JNK3-N-Tat 1 hr before MPP^+^ (100 μM) treatment resulted in significant attenuation of cell death. We also investigated the effects of the JNK3 peptides on MPP^+^-induced neuronal death by immunostaining for MAP2, which is specific to neurons. Statistical analysis indicated that JNK3-N-Tat pretreatment reduced the loss of MAP2-positive neurons (Fig 6G and Fig 6H).

Immunostaining for the marker TH was performed to determine whether JNK3-N-Tat plays a protective role against MPTP-induced dopaminergic neuronal death. Compared with sham-treated normal control mice, mice that were administered saline vehicle before each MPTP injection exhibited a marked reduction in the number of TH-positive neurons in the SNc (Fig 6B.ii) and decreased dopaminergic axon density in the striatum (Fig 6C.ii). In contrast, JNK3-N-Tat administration reduced the loss of TH-positive neurons in the SNc (Fig 6B.iii) and attenuated dopaminergic terminal loss in the stratum (Fi 6C.iii). JNK3-4A-Tat administration did not confer any protection against MPTP-induced dopaminergic neuronal injury (Fig 6B.iv and Fig 6C.iv). Therefore, the JNK3-inhibitory peptide JNK3-N-Tat exerted protective effects against MPTP-induced dopaminergic neuronal injury.

## Discussion

A growing body of evidence suggests that the JNK3 signaling pathway acts in a pro-apoptotic manner during the pathogenesis of PD. It has been suggested that the neuron-specific isoform of JNK3 is involved in apoptosis [19,20]. A variety of studies, particularly genetic knockout studies, have uncovered that JNK3 plays a pivotal role in the death of adult neurons. Yang et al. reported that hippocampal neurons are highly resistant to kainic acid-induced cell death in JNK3-/- or c-Jun mutant mice [20]. In JNK3 knockout mice, β-amyloid-induced death is attenuated in cortical neurons [21]. The study by Kuan et al. showed that a targeted deletion of JNK3 reduces stress-induced JNK activity and protects mice against neuronal apoptosis [19]. Examination of various JNK-deficient mice revealed that only 15% of TH-positive cells were lost in JNK2/3 null mice, suggesting that both JNK2 and JNK3 are required for MPTP-induced dopaminergic cell death *in vivo* [22]. A JNK3 knockout conferred persistent protection in SNc neurons and also provided transient protection against 6-OHDA-induced injury [23]. Ries et al. reported that apoptosis was completely abrogated and that the survival of dopaminergic neurons was extended in homozygous JNK2/3 double-null-mutant animals [24]. Consistent with the above studies, our results demonstrate that the activation of JNK3 and the binding of β-arrestin2 to JNK3 are dramatically increased in MPP^+^-treated cells, suggesting that JNK3 plays an essential role in mediating neuronal apoptosis in PD.

As the JNK3 signaling pathway is critical to neuronal death, JNK3 has garnered considerable interest as a potential therapeutic target [25]. Of all the JNK inhibitors, SP600125 and CEP1347 have been best studied. SP600125, a reversible ATP-competitive JNK inhibitor, was reported to inhibit JNK with high potency and high specificity [26]. Moreover, SP600125 conferred protection to dopaminergic neurons against MPTP-induced injury [27,28]. However, SP600125 was also reported to have unpredictable side effects. SP600125 acts as an inhibitor with a similar potency to all three JNK isoforms, and it can inhibit the activation of c-Jun in a dose-dependent manner [29]. Upstream MAP kinases, such as MKK3, MKK4, MKK6 and MKK7, were also inhibited by SP600125 [26]. CEP-1347, an inhibitor of MLK kinases, also displayed neuroprotective effects in an MPTP treatment model [30]. However, in 2007, CEP-1347 treatment was demonstrated to be ineffective for patients with early stage PD [31]. Therefore, considering the importance of JNK3 and the failure of MLK and JNK inhibitors to treat PD, identifying potent specific inhibitors of JNK3 may allow for the protection of dopaminergic neurons in PD.

Involvement of the ASK1-JNK3 pathway in PD models suggests a possible target for the prevention of dopaminergic neuronal death in PD. β-arrestin2, a member of the JNK scaffold family, is a crucial mediator of ASK1-JNK3 signaling pathway activation. In particular, the interaction between the N-terminus of JNK3 and β-arrestin2 may represent a key step in this process. A functional peptide, termed JNK3-N-Tat, was designed to mimic the N-terminus of JNK3. Here, we present evidence that this peptide inhibits the access of β-arrestin2 to its target domain in JNK3, thereby inhibiting the activation of JNK3 in both cellar and mouse models of PD. Based on the above results, we tested whether administration of this peptide could prevent dopaminergic neuron degeneration following MPP^+^/MPTP insult. Our study demonstrates that the JNK3-N-Tat peptide protects dopaminergic terminals and SNc cell bodies against degeneration. Our study also showed (Fig 6) JNK3-N-Tat peptide protected dopaminergic axonal and JNK3 may also have a beneficial important role for axonal protection in models of PD, Consistent with Cheng et al study [32]. We have found this interesting phenomenon. Research about related axonal were carried out.The downstream mechanisms responsible for the protective effects of JNK3-N-Tat require further characterization. Activated JNK3 phosphorylates the transcription factor c-Jun, which may in turn enhance AP-1 transcriptional activity to modulate gene expression [33] and apoptotic processes [34]. This study indicates that treatment with JNK3-N-Tat attenuates the aberrant phosphorylation of c-Jun induced by MPP^+^/MPTP administration. Moreover, our results showed that JNK3-N-Tat treatment resulted in the retention of JNK3 in the cytoplasm in a cellar model of PD. These results suggest that the nuclear signaling pathway mediated by JNK3 activation is altered by JNK3-N-Tat treatment. As activated JNK3 also can promote apoptosis and cell death by regulating the mitochondrial activation, we measured mitochondrial function and morphology. We show that JNK3-N-Tat treatment protected mitochondria against MPP^+^-induced neuronal injury, and flow cytometry revealed that JNK3-N-Tat treatment attenuated MPP^+^-induced neuronal apoptosis. These results suggest that the mitochondria-dependent apoptotic pathway mediated by JNK3 activation is also involved in dopaminergic neuronal apoptosis induced by MPP^+^/MPTP and that treatment with JNK3-N-Tat inhibits mitochondria-dependent apoptosis. However, the cortical neurons lack the mesencephalic dopamine transporter (DAT) and so are not good model to study MPP^+^. The study of primary midbrain cultures may give more evidence in our further study

Taken together, we conclude that JNK3-N-Tat can downregulate the activation and nuclear translocation of JNK3 and may function to inhibit transcription factor activity and protect mitochondria to attenuate MPP^+^/MPTP-mediated damage. However, other mechanisms, including oxidative and nitrosative stress, the accumulation of aberrant or misfolded proteins, and ubiquitin proteasome system dysfunction, may also be critical to the pathogenesis of sporadic and familial forms of PD. For example, oxidative insults cause mitochondrial dysfunction, which is a major trigger of apoptosis. Therefore, we must await further evidence demonstrating the effects of JNK3-N-Tat on mitochondrial function and apoptotic processes in naturally occurring PD.

In summary, several lines of evidence presented in this study clearly support the activation of JNK3 *in vitro* in an MPP^+^-induced cellar model of PD and that the cell-permeable JNK3-N-Tat peptide inhibits this activation by disrupting the binding of β-arrestin2 to the N-terminus of JNK3, thereby exerting protective effects against MPTP-induced dopaminergic neuronal toxicity. Our results suggest that this peptide may be useful in therapeutic strategies for PD. However, the potential protective effects of JNK3-N-Tat on dopaminergic neurons require further investigation.

## Supporting Information

S1 Fig (DOCX)

S2 Fig (DOCX)

S3 Fig (DOCX)
