# Supplementary figures and images for: Small Peptide Inhibitor of JNK3 Protects Dopaminergic Neurons from MPTP Induced Injury via Inhibiting the ASK1-JNK3 Signaling Pathway
Source: PLoS One. 2015 Apr 9;10(4):e0119204. doi: 10.1371/journal.pone.0119204 (PMC4391862; doi:10.1371/journal.pone.0119204)

**
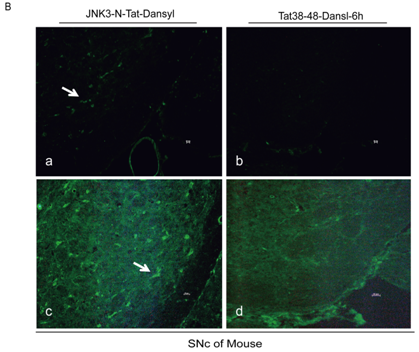
Figure** **S1**

Supplement: S1 Fig — (DOCX) [file pone.0119204.s001.docx]

**Figure S1.**

A


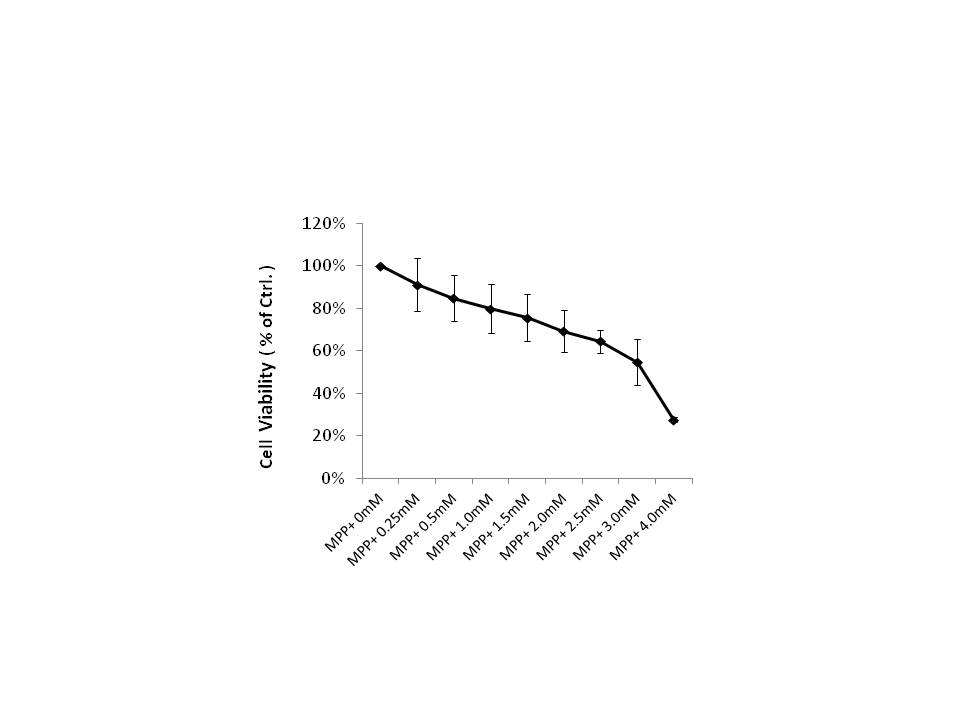


**

**

**

*

*

B


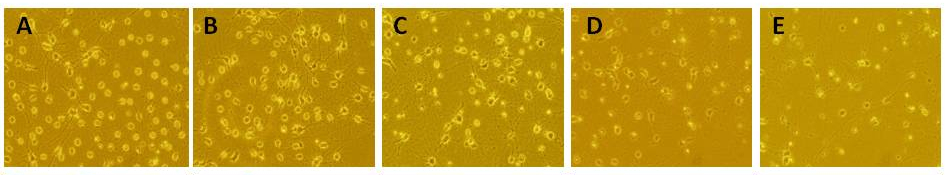

Supplement: S2 Fig — (DOCX) [file pone.0119204.s002.docx]

**
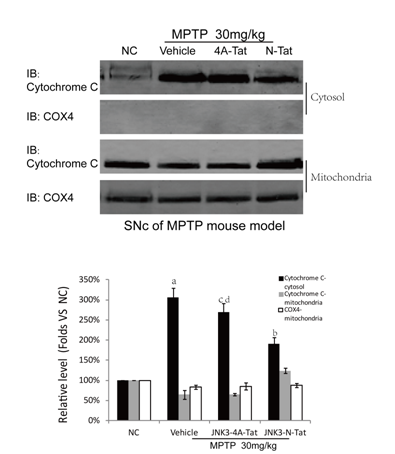
Figure S3**

Supplement: S3 Fig — (DOCX) [file pone.0119204.s003.docx]
